# Diagnostic value of high-resolution ultrasonography and computed tomography in the diagnosis of nasal bone fracture

**DOI:** 10.12669/pjms.41.9.12706

**Published:** 2025-09

**Authors:** Xiaoyun Yang, Yi Wang, Yujin Feng, Lijuan Gao

**Affiliations:** 1 Xiaoyun Yang, Department of Abdominal Ultrasound, Second Hospital of Hebei Medical University, Shijiazhuang, Hebei Province 050000, P.R. China; 2 Yi Wang, Department of Abdominal Ultrasound, Second Hospital of Hebei Medical University, Shijiazhuang, Hebei Province 050000, P.R. China; 3 Yujin Feng, Department of Abdominal Ultrasound, Second Hospital of Hebei Medical University, Shijiazhuang, Hebei Province 050000, P.R. China; 4 Lijuan Gao, Department of Imaging, Second Hospital of Hebei Medical University, Shijiazhuang, Hebei Province 050000, P.R. China

**Keywords:** High-resolution ultrasonography, Computed tomography, Different types, Nasal bone fracture

## Abstract

**Objective:**

To compare the diagnostic abilities of high-resolution ultrasonography (HRUS) and computed tomography (CT) in different types of nasal bone fractures (NBFs).

**Methodology:**

Clinical data of patients with suspected NBFs who were examined in the second hospital of Hebei Medical University from April 2023 to April 2025 were retrospectively analyzed. All patients underwent HRUS and CT examination. Based on the results of CT examination and the characteristics of ultrasound images, NBFs were characterized as: simple linear fracture, comminuted fracture, compound fracture and incomplete fracture.

**Results:**

According to the CT examination, out of 100 cases of suspected NBFs, there were 38 cases of simple linear fractures, 18 cases of comminuted fractures, 11 cases of compound fractures, four cases of incomplete fractures and 29 cases of undetected NBF. HRUS identified 41 cases of simple linear fractures, 19 cases of comminuted fractures, nine cases of compound fractures, four cases of incomplete fractures and in 27 cases, NBFs were not detected. The detection rates of HRUS and CT examination were 73.0% and 71.0%, respectively, with no significant difference in diagnostic ability (P > 0.05).

**Conclusions:**

HRUS and CT have similar efficacy in diagnosing different types of nasal bone fractures(NBFs.)

## INTRODUCTION

The anatomical structure of the nose, which includes bilateral nasal bones and the frontal process of the maxilla, is considered one of the most vulnerable parts of the facial bones, susceptible to traumatic fractures.^1^,^2^ Nasal bone fractures (NBFs) are the most common craniofacial bone injuries, accounting for over 40% of all cases of facial fractures.^3^

A standardized diagnosis of NBF requires a detailed examination of the lateral wall, nasal dorsum, nasal septum and other parts.^1^,^2^,^4^ While physical evaluation is an integral part of NBF diagnosis, the presence of soft tissue edema and hematoma around the nasal bone may interfere with the physical determination of fracture, increasing the risk of misdiagnosis.^5^ Therefore, a comprehensive diagnostic evaluation of facial injuries requires imaging as an auxiliary examination method. However, the reliability of the plain film radiographs, routinely used in the cases of NFSs, is very limited,^6^ especially in patients with cortical fractures, fractures of the nasal septum, displacement of the fracture, or the adjacent bone fracture.^7^ Computer tomography (CT) has long been the most widely used method for diagnosing NBF.^8^ However, this imaging technique is expensive, may lead to adverse effects due to the use of ionizing radiation and therefore does not apply to certain groups (such as pregnant women, patients with cervical spondylosis and patients with poor cooperation).^9^

High-resolution ultrasound (HRUS) is a non-invasive imaging method, efficiently used for diagnosing NBFs.^10^ Studies have shown that ultrasound has the same sensitivity and specificity as CT, with high accuracy in detecting maxillofacial fractures but without the hazards associated with ionizing radiation.^4^,^9^ Furthermore, HRUS produces real-time dynamic images, showing the continuity of cortical bone, fracture mode, degree of soft tissue injury, vascular perfusion, etc. and thus provides comprehensive information for clinicians before surgery.^10^,^11^ Compared with the traditional CT examination, HRUS is convenient, fast and cost-effective, making it a method of choice in settings with limited resources.^12^ However, there is still insufficient evidence for the comparative diagnostic value of CT and HRUS in different fracture morphologies and displacement directions. This study aimed to evaluate and compare the diagnostic abilities of CT and HRUS in identifying different types of NBFs.

## METHODOLOGY

Clinical data of patients with suspected NBFs, who were examined in the second hospital of Hebei Medical University from April 2023 to April 2025, were retrospectively analyzed. All patients underwent HRUS and CT examination within 24 hours of trauma. HRUS was performed first, followed by a CT examination within two hours.

### Ethical Approval:

The research ethics committee of the Second Hospital of Hebei Medical University approved this observational study with the number 2020-R132, Date: March 3, 2020.

### Inclusion criteria:

- Clinical symptoms and signs highly suggestive of NBF.
- Have one of the following symptoms: noticeable nasal swelling, nasal congestion, nosebleed or nasal type change.
- First time nasal trauma, no previous history of nasal trauma.
- Age 18-80 years old.
- Clear mechanism of injury; the cause of injury is foreign body impact or a traffic accident.
- The image data are complete.

### Exclusion criteria:

- Previous history of nasal surgery or fracture.
- Nasal tumors and chronic nasal diseases.
- Patients with mental illness or cognitive impairment and other reasons that prevent cooperation.
- Accompanied by fractures in other parts of the skull and face.

### Imaging:

### HRUS:

Philips epiq5 color Doppler ultrasound system was selected with an 18 MHz high-frequency linear array probe and the frequency range of 5~18 MHz. The patient was instructed to lie on their back with the head slightly tilted back. Sterile coolant was applied to the probe and the doctor performed the scanning, ensuring that no pressure was applied to the affected part. The main anatomical structures of the nasal bone and alar cartilage were scanned by the method of combining cross-section and longitudinal section. Scanning focused on observing the continuity of the nasal cortical echo, looking for cortical interruption to determine the fracture. The displacement of the fracture end, the shape of the fracture end, the thickness of the subperiosteal hematoma and the changes of the surrounding soft tissue were assessed. The local blood flow perfusion of the fracture and the blood flow spectrum characteristics of the adjacent blood vessels were observed by color Doppler. The inspection image was recorded and stored digitally for further observation and analysis. The imaging lasted no more than five minutes per person.

### CT:

Philips 16-row spiral CT was used for volume scanning. The patients were positioned on their backs on the fixed head restraint so that the midsagittal plane was parallel to the center line of the frame. The scanning range of the skull was from the superior orbital margin to the apex of the alveolar process and enveloped the entire nasal bone and its adjacent facial bone structure. The spiral scanning mode was adopted and the scanning parameters were as follows: tube voltage 120 kV, tube current 175 mA, collimator size 16 × 0.75 mm, layer thickness and layer spacing 1.0 mm, pitch 0.44, frame speed 0.5 s/R, FOV 180 mm, matrix size 512 × 512. A high-resolution bone algorithm combined with edge enhancement technology was used to improve the display effect of cortical bone and the sensitivity of fracture detection. The post-processing work included multiplanar reconstruction with a reconstruction layer thickness of 1-2 mm; the axial reconstruction took the auditory orbital line as the baseline and the coronal reconstruction was parallel to the long axis of the nasal bone. Complex fractures required 3D volume reconstruction to judge the spatial relationship between fractures. All images were analyzed offline. According to the diagnostic criteria of fracture in ultrasonic diagnostics -2^nd^ Edition and recent classification systems,^13^ NBFs were divided into the following four types:

- ***Simple linear fracture:*** Single non-displaced fracture line.
- ***Comminuted fracture:*** Multiple fracture lines with fragmentation.
- ***Compound fracture:*** Fracture with displacement or angulation.
- ***Incomplete fracture:*** Partial cortical disruption without complete separation. All operations and diagnoses are performed by four doctors who have worked for more than eight years. All disagreements were resolved by consultation. To minimize operator dependence, all four operators completed standardized training and differences were resolved through consultation to ensure the consistency of diagnosis.

### Statistical analysis:

The data were analyzed by SPSS 26.0 statistical software (IBM Corp, Armonk, NY, USA). Post-hoc power analysis confirmed that there was sufficient statistical efficacy (> 80%) to detect the clinical significance difference between imaging methods. Age and body mass index (BMI) were reported as mean and standard deviation (SD). The results of CT and HRUS in the diagnosis of NBF were statistically analyzed by a four-grid table chi-square test. P<0.05 indicated significance.

## RESULTS

One hudnred patients with clinically suspected NBFs were included in this study. All patients completed HRUS and CT examination. There were 62 males and 38 females in the cohort, with an average age of 36.94 ± 8.35 years. Among them, 78 NBFs (78.0%) were caused by foreign body impact and 22 fractures (22.0%) were caused by traffic accidents (Table I).

**Table-I:** Baseline characteristics of patients.

Baseline characteristics		n=100	Proportion (%)
Gender	Male	62	62.0
	Female	38	38.0
Cause of injury	Foreign body impact	78	78.0
	Traffic accident	22	22.0
Age (years)		36.94 ± 8.35	-
BMI (kg/m²)		23.52 ± 1.58	-

BMI: body mass index.

**Fig.1:**
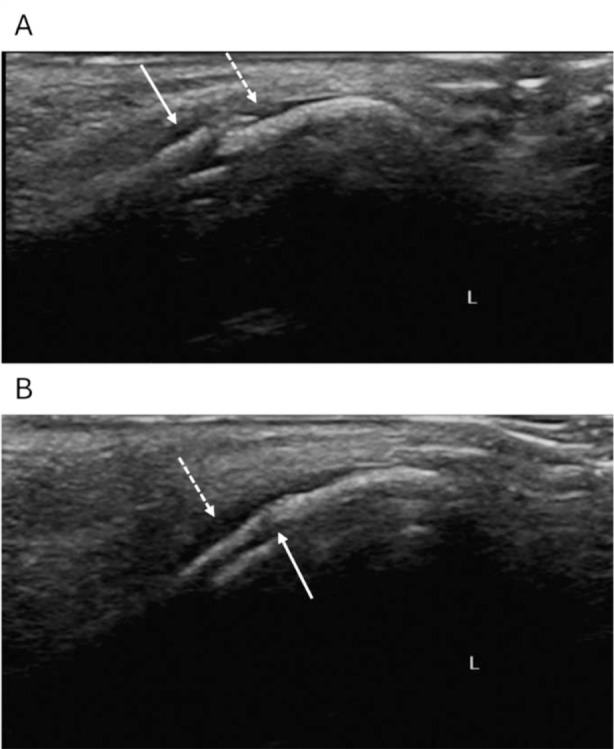
Ultrasound image of a 22-year-old male patient with simple linear nasal bone fracture. Pain and swelling of the right orbit after blunt trauma. A: The hyperechoic zone of bone cortex is interrupted continuously and separated by dislocation (solid line arrow) and a small patchy anechoic area can be seen around the fracture (dotted line arrow). B: After reduction of nasal bone fracture, the fracture is well aligned (solid arrow) and only a narrow band of anechoic area (dotted arrow) can be seen around it.

**Fig.2:**
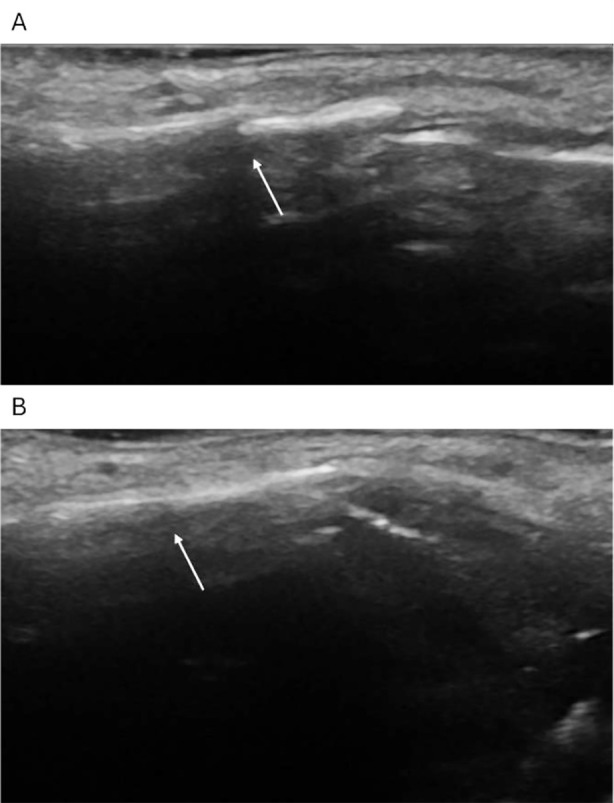
Ultrasound image of a 42-year-old female patient with comminuted nasal bone fracture. A: The hyperechoic zone of bone cortex was interrupted continuously and separated by dislocation. The swelling of soft tissue around the fracture was not obvious. B: After reduction of nasal bone fracture, the fracture ends were well aligned.

**Fig.3:**
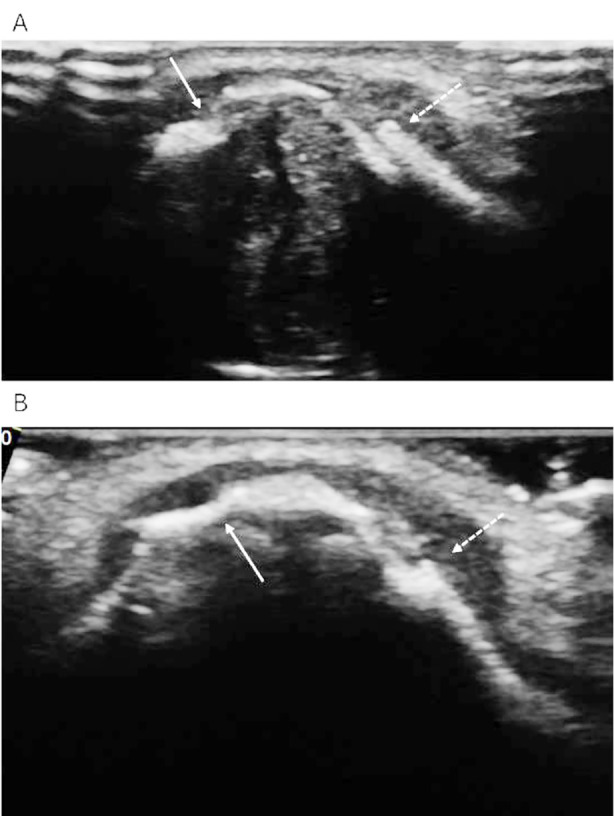
Ultrasound image of a 51-year-old male patient with compound nasal bone fracture. A: Partial angular displacement of the nasal bone, no dislocation of the broken end (solid line arrow), continuous interruption of the hyperechoic zone of the bone cortex, dislocation and separation of the broken end, overlapping each other (dotted line arrow). B: After reduction of nasal bone fracture, the fracture ends were well aligned (solid arrow), the fracture ends were moderately aligned and the periosteum was rough (dotted arrow).

**Fig.4:**
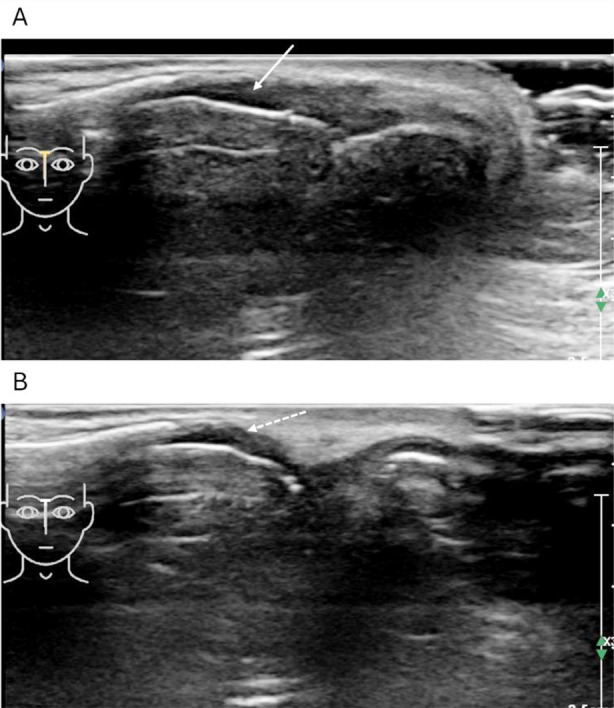
Ultrasound image of a 44-year-old male patient with incomplete fracture. A: The hyperechoic zone in the bone cortex of the nasal bone is continuous, slightly angled without displacement. Irregular and uneven echo areas can be seen in the superficial subcutaneous soft tissue (solid arrow). B: Two weeks later, the reexamination showed that the hyperechoic zone in the bone cortex of the nasal bone was continuous, slightly angled without displacement and the uneven echo area of the superficial subcutaneous soft tissue disappeared (dotted arrow).

CT examination results of patients with suspected NBF identified 71 cases as positive and 29 cases as negative for NBF, while HRUS detected 73 fracture-positive and 27 fracture-negative cases. The proportions of different types of NBFs are shown in Table II. Simple linear fracture was the most common type in the two examination methods, accounting for 53.5% (38/71) in CT examination and 56.2% (41/73) in HRUS (Fig.1). The proportions of comminuted fractures in CT and HRUS were 25.4% (18/71) and 26.0% (19/73), respectively (Fig.2). The detection rate of complex fractures in CT and HRUS were 15.5% (11/71) and 12.3% (9/73), respectively (Fig.3). The detection rate of incomplete fracture in the two methods was 5.5% (Fig.4). Chi square test showed that there was no significant difference between the two methods in judging the type of fracture (*χ^2^*=0.847, P=0.838).

**Table-II:** Comparative analysis of diagnostic efficacy of HRUS and CT, n(%).

Methods	Positive					Negative	Detection rate (%)
	Simple linear	Comminuted	Complex	Incomplete fracture	Total		
CT	38(53.5)	18(25.4)	11(15.5)	4(5.6)	71(71.0)	29(29.0)	71.0
HRUS	41(56.2)	19(26.0)	9(12.3)	4(5.5)	73(73.0)	27(27.0)	73.0

## DISCUSSION

This study retrospectively analyzed the HRUS and CT imaging data of one hundred patients with suspected NBFs and demonstrated that the two methods have comparable diagnostic value. The detection rate of HRUS was 73.0% and the detection rate of CT examination was 71.0% for different types of NBFs. A fixed HRUS-CT sequence may introduce bias. However, the two-hours interval limits the soft tissue changes and the blind interpretation by different operators minimizes this effect. Randomized image sequences should be considered in future research.

While the detection rate of HRUS for simple linear NBF was slightly higher than that of CT, there was no significant difference between the two methods in identifying different fracture types. These results confirm previous reports that demonstrated the efficiency of HRUS in detecting bone diseases, including fractures.^14^,^15^ A study by Lee et al.^10^ found that the accuracy of HRUS, CT and conventional radiography in detecting nasal fractures was 100%, 92.1% and 78.6%, respectively. Lee et al.^10^ further confirmed that although CT showed higher sensitivity and specificity than HRUS or X-ray examination, HRUS findings were helpful to evaluate the midline of the nasal bone, which is consistent with the results of this study. At the same time, this study found a similar diagnostic ability of HRUS and CT for different types of NBFs. This observation has important clinical significance. The most crucial advantage of HRUS technology is the absence of exposure to ionizing radiation. Even though the radiation dose of modern CT scanning has been reduced to the minimum safe level, special categories of patients, such as pregnant women and children, as well as patients who require multiple reviews, still face potential health risks caused by cumulative radiation exposure.^14^-^17^ The results of this study confirm the diagnostic value of a safer HRUS for patients with different types of NBFs.

This study found no significant difference in diagnostic accuracy between ultrasound and CT. The detection rate of HRUS and CT was 73.0% and 71.0%, respectively. Additionally, the results of HRUS and CT were consistent across patients with different fracture types, further confirming equal sensitivity and specificity of HRUS and CT.^18^,^19^ Ultrasound can effectively detect simple linear fractures, clearly demonstrate the continuity and interruption of cortical bone and more accurately identify minor cortical defects.^20^,^21^

This study showed that the proportion of simple linear fractures detected by HRUS was 56.2%, which was slightly higher than the detection rate of CT (53.5%). The result confirms the value of ultrasound in diagnosing and treating minor fractures.^22^,^23^ Moreover, HRUS can achieve real-time dynamic imaging and make accurate judgments on changes at specific fracture sites and soft tissue, therefore facilitating the observation of subperiosteal hematoma.^24^,^25^ However, it is important to note that ultrasound examination has certain limitations, such as variability due to the examination quality, which is determined by the technical level of its operators, clinical experience and other factors.^26^ Since ultrasound examination is highly dependent on the technical skills of the operator, hospitals need to establish a comprehensive training mechanism and quality control system to ensure the accuracy of relevant diagnoses and treatment work. Additionally, for complex comminuted fractures, CT examination can provide a clearer view of the three-dimensional situation of the fractures and the spatial distribution of bone fragments, which is conducive to preoperative planning and establishing standard operating procedures.^11^

Based on the above findings, the hierarchical diagnostic workflow should rely on ultrasound evaluation as a pre-screening method, primarily used in the emergency department to facilitate rapid assessment. In all complex cases of difficult diseases of unclear HRUS results, CT examination should be carried out to obtain more accurate anatomical image information.^12^,^27^ Such a diagnostic and treatment approach not only ensures the accuracy but also makes rational use of the respective advantages of the two technologies, especially in cases with complex anatomical structures that require specific skills, best scanning angles and depths. HRUS can also be used to dynamically monitor the surrounding soft tissue and evaluate the formation process of hematoma, the severity of tissue edema and other changes. And we suggest that HRUS should be used as the initial emergency screening for suspected NBF and CT should be used for uncertain cases, complex fractures, or surgical planning. This method reduces radiation exposure while maintaining the accuracy of diagnosis. This information has significant reference value for the development of clinical treatment.^4^,^5^,^28^ detection rate.

### Limitations:

It includes small sample size which limits the subgroup analysis and universality; Moreover, due to the lack of intraoperative confirmation, fracture diagnosis was made solely based on imaging and lack of long-term follow-up did not allow to evaluate the clinical outcome and fracture healing. Other limitations are that there was no age-specific analysis of different populations; besides it is a single center retrospective design of the study and Fixed imaging sequence may have introduced bias; In addition, despite standardization efforts, there is still operator dependence and there was no formal Kappa statistical inter-observer reliability evaluation, which limits the reproducibility of the evaluation.

## CONCLUSION

HRUS and CT have similar diagnostic ability for different types of NBFs. HRUS shows the same diagnostic performance as CT and can be used as a valuable alternative method for NBF diagnosis, especially for radiation-sensitive people.

### Authors’ contributions:

**XY:** Study design, literature search and manuscript writing.

**YW, YF and LG:** Data collection, data analysis and interpretation. Critical Review

**XY:** Manuscript revision and validation and is responsible for the integrity of the study.

All authors have read and approved the final manuscript.
